# Children and innovation: play, play objects and object play in cultural evolution

**DOI:** 10.1017/ehs.2021.7

**Published:** 2021-02-05

**Authors:** Felix Riede, Matthew J. Walsh, April Nowell, Michelle C. Langley, Niels N. Johannsen

**Affiliations:** 1 Department of Archaeology and Heritage Studies, Aarhus University, Moesgård Allé 20, 8270 Højbjerg, Denmark; 2 Interacting Minds Centre, Aarhus University, 8000 Aarhus C, Denmark; 3 Department of Ethnography, Numismatics, Classical Archaeology and University History, Museum of Cultural History, University of Oslo, 0164 Oslo, Norway; 4 Department of Anthropology, University of Victoria, Victoria, British Columbia, Canada; 5 Australian Research Centre for Human Evolution, Griffith University, Brisbane, Australia; 6 Forensics and Archaeology, School of Environment and Science, Griffith University, Brisbane, Australia

**Keywords:** Playthings, pedagogy, cultural evolution, human evolution, niche construction

## Abstract

Cultural evolutionary theory conceptualises culture as an information-transmission system whose dynamics take on evolutionary properties. Within this framework, however, innovation has been likened to random mutations, reducing its occurrence to chance or fortuitous transmission error. In introducing the special collection on children and innovation, we here place object play and play objects – especially functional miniatures – from carefully chosen archaeological contexts in a niche construction perspective. Given that play, including object play, is ubiquitous in human societies, we suggest that plaything construction, provisioning and use have, over evolutionary timescales, paid substantial selective dividends via ontogenetic niche modification. Combining findings from cognitive science, ethology and ethnography with insights into hominin early developmental life-history, we show how play objects and object play probably had decisive roles in the emergence of innovative capabilities. Importantly, we argue that closer attention to play objects can go some way towards addressing changes in innovation rates that occurred throughout human biocultural evolution and why innovations are observable within certain technological domains but not others.

## Graphical abstract

**Figure:**
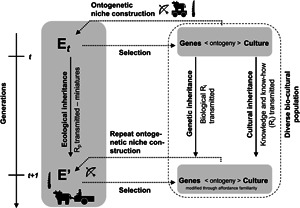

**Social media summary:** Niche construction theory predicts that letting small humans play with toys may make them more likely to innovate

## Introduction

In late 2019, 15 researchers in archaeology, anthropology, primatology and psychology from across the globe came together in Brisbane (Australia) to debate four questions:

1. Throughout human evolution, what roles might children have played in the socioeconomic lifeways of the communities in which they lived?
2. Could children be a primary driver for dynamic changes in technology in prehistory – particularly over the past 300,000 years?
3. How can we use data on recent human and primate subadults to learn about those from millennia ago?
4. What could these patterns of past child-centred innovation tell us about the role of children in the present?

The papers which form this special collection – ‘Children and Innovation’ – present some of the results of that Wenner–Gren Workshop, and we offer here an introductory paper which touches on many of the aspects discussed during the meeting.

In recent years, evolutionary approaches have made considerable headway in understanding the patterns and processes of culture change (e.g. Lipo et al., 2006; Mace et al., 2005; Mace & Holden, 2005; O'Brien, 2008; Shennan, 2009). Casting culture as a multigenerational system of information transmission has facilitated the formal modelling and empirical interrogation of how cultural traditions change over time and under different regimes of social learning (cf. Rendell et al., 2011). In this context, it has been pointed out that humans have a derived sense of pedagogical awareness: both teaching and being taught are essential features of what allows *Homo sapiens* to accumulate the astounding array and diversity of cultural competences and technologies that characterise at least the last 300,000 years of human biocultural evolution (e.g. Castro & Toro, 2014; Csibra & Gergely, 2009; Gärdenfors & Högberg, 2017; Kline, 2015; Tehrani & Riede, 2008 and many others). Quantitative modelling, too, has suggested that, for culture to become cumulative, social learning including active teaching seems requisite (Dean et al., 2014; but see Reindl et al., 2020). In fact, Nowell (2021), has emphasised the importance of oral storytelling as a pedagogic tool in foraging societies.

Some anthropologists field somewhat opposing views, arguing that formal teaching is largely unimportant within many traditional societies. Based on extensive ethnographic assessments, Lancy (2010, 2016) and MacDonald (2007), for instance, maintain that in many small-scale societies – and especially amongst foragers – children are efficient autodidacts, acquiring technical and procedural competences through trial and error, with virtually unregulated contact with adult material culture or explicit observation of adults utilising it. By the same token, it is clear from numerous cross-cultural surveys that children play games – often involving ad hoc or even specially manufactured play objects – that emulate adult activities and paraphernalia (e.g. Bloch, 1989; Ember & Cunnar, 2015; Langley & Litster, 2018; Pellegrini & Bjorklund, 2004). Within these contexts, children often appear to innovate spontaneously (Neldner et al., 2020), although other studies also show that children's engagement with objects changes as they age (Alessandroni, 2020; Vig, 2007). Still, in some ethnographic settings – and mostly in sedentary societies and for some key technologies – fairly strict apprenticeship regimes are documented (e.g. Stout, 2002). In addition, given the evidence for teaching in the archaeological record (Tehrani & Riede, 2008), it remains unclear to what degree free learning was the norm in early human populations and when any major transitions from primarily trial-and-error learning to instruction may have occurred (Tennie et al., 2017).

Models of material culture change suggest that, in the absence of teaching, variation is likely to be introduced indiscriminately (Eerkens & Lipo, 2005, 2007). Under such conditions, the remarkably stable traditions of manufacture and use documented in the archaeological record might not have emerged. While strong teaching conditions would be predicted to stifle innovation, a prevalence of trial-and-error learning would conversely be predicted to only rarely lead to innovations that truly improve on previous designs (Walsh et al., 2019), especially if we also accept that humans are in fact not all that good at predicting the future (Mesoudi, 2008).

A recent review of the generative processes and sources of variation in culture vividly demonstrates that no unequivocal understanding of ‘guided variation’ has been reached (Mesoudi, 2021). At the same time, none of the mechanisms addressed in that review substantially heed the inter-generational effects of material culture on the cognitive propensities for domain-specific innovation. Importantly, innovations are associated with risk of failure as well as investments in time and resources, which could instead have been directed towards essential activities such as food-getting or reproduction. In sum, explaining the mechanism behind salient innovation in pre-modern societies remains elusive – especially in those societies of the deep past where dedicated craft specialists were rare or absent.

In the laboratory, cultural evolution experiments commonly involve the transmission of knowledge from adult to adult and where prior exposure to a given technology is *minimised* (e.g. Caldwell & Millen, 2008; Derex et al., 2019). In modelling studies, innovation has commonly been likened to random mutation, reducing its occurrence to chance or fortuitous transmission error: ‘Cultural innovation is to cultural evolution what mutation is to biological evolution: without innovation, cultural traits and therefore cultural transmission would not exist’ (Lehmann et al., 2010: 2356). The issue of how innovation can be defined has been tackled repeatedly (Carr et al., 2016; Hoffecker, 2012; Shennan, 1989), and most recently by Walsh and colleagues (2019). Supplementing Carr et al. (2016) we here argue – with reference to two archaeological case studies – that pitching random innovation, whether in the form of true novelty or novel combinations, against full causal understanding ignores the familiarity and hence cognitive priming obtained by children growing up in niches furnished with material culture that can include play objects.

We here attempt to reconcile the opposing notions that, on the one hand, innovations are random occurrences, while on the other, innovations are acts of conscious, goal-oriented manipulation towards a premediated outcome. We do so by combining insights from hominin life-history with archaeological observations on play object provisioning under the umbrella of niche construction theory. In particular, we focus on complex technologies made up of multiple components, the combined functionality of which is not inherent in any of the constituent parts. After briefly introducing the niche construction perspective, we review pertinent findings from ethology, developmental psychology and anthropology and then discuss how archaeological proxies – via case studies from Greenland prehistory and the Eurasian Neolithic – can be used to illustrate how technological innovation can be primed during ontogeny through play object provisioning. We here focus specifically on two dimensions – one material and the other cognitive – of such ontogenetic niche construction: (1) the role of functional miniatures and their role in innovation *within particular technological domains* (i.e. specific technologies); and (2) how associative or analogical reasoning can work with play objects to facilitate innovations that reach *across domains* (e.g. from technology to cosmology) in what Sterelny (2003) termed downstream epistemic engineering (see also Wheeler & Clark, 2008).

## Ontogenetic niche construction, object play and play objects

**Figure 1.**
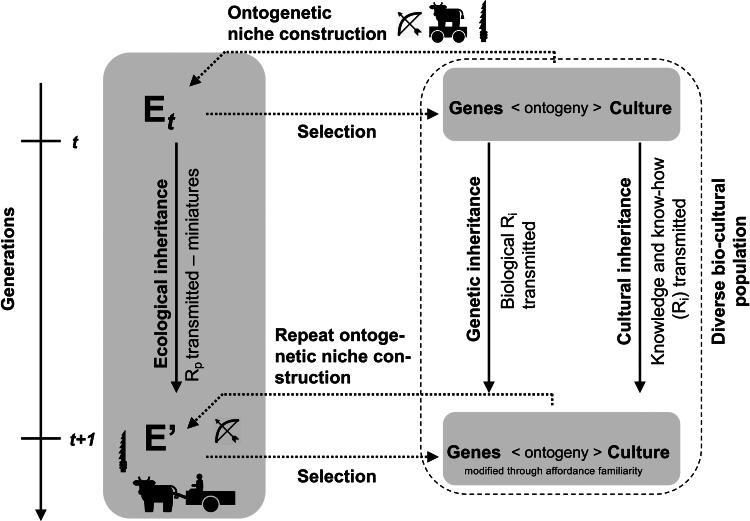
The three domains of inheritance of niche construction theory: genetic, cultural, and ecological with the respective resources (R_p_, R_i_) that are transferred. Redrawn and adapted from Odling-Smee (2007).

Niche construction theory posits that not only genetic and cultural information is passed on from generation to generation but that also environmental modifications are inherited. This process is not only true in *Homo*, albeit peculiarly and extensively so in this lineage (Odling-Smee et al., 2003). Anthropologists have long argued that material culture constitutes an ‘extra-somatic means of adaptation’ (Binford, 1962: 218), a notion that goes back to at least Leslie White (1959) and, loosely, V. Gordon Childe (1936), and which presaged Dawkins’ (1982) extended phenotype. The idea of the extended phenotype re-entered the anthropological discussion in the context of the nascent evolutionary archaeological paradigm of the 1990s (O'Brien & Holland, 1995). While the tight linkage between genotype and extended phenotype turned out to be less useful for investigations of human culture – with perhaps the exception of its earliest variants (Corbey et al., 2016) – it did become clear that the actions of organisms on the environment, via their extended phenotypes, critically modify those organisms’ physiological niche parameters. Critically, the longevity of some of these modifications, often across multiple generations, entails selection-modifying legacies. As new members are born into a niche-constructing population, the modified niche components and its resources become ‘ecologically inherited’ (Figure 1).

Niche modifications can target the ontogenetic environment or act on environmental components that mollify and direct selection; they can buffer organisms against environmental changes and so create adaptive lags (Laland & Brown, 2006) or create entirely novel interactions that can result in further behavioural or genetic change. A final important feature of niche construction is that such niche modifications can have unintended positive or negative selective effects in the long term (see Riede, 2019 for a recent review).

The majority of studies concerned specifically with human niche construction have focused on model systems such as animal and plant domestication (e.g. Altman & Mesoudi, 2019; Bentley & O'Brien, 2019; Boggs, 2016; Boivin et al., 2016; Fox et al., 2017; Zeder, 2016). However, niche construction can also operate at a more intimate scale and with effects not so much on other species but on individuals belonging to the niche-constructing species itself. During niche construction purely informational resources (R_i_) as well physical resources (R_p_) can be transmitted (Figure 1). Physical resources, such as artefact structures, themselves will often also contain semantic cues (Jeffares, 2010).

That the ontogenetic niche is especially important in humans is well established. *Homo sapiens* newborns are helpless and take a long time to mature; infant and childhood survival demand considerable care, which in obligate tool-users results in a rich array of trappings such as child-carrying devices, cots, slings and toys. The criticality of this ontogenetic niche is also appreciated in the context of cumulative cultural evolution. Tomasello (1999: 512) asserts: ‘The major part of the ratchet in the cumulative cultural evolution of human societies takes place during childhood. That is, each new generation of children develops in the “ontogenetic niche” characteristic of its culture … mastering the artifacts and social practices that exist at that time’ (see also Tomasello, 2020). However, children are not simply empty vessels for adult culture. Increasingly children's agency is seen as a key force in human niche construction (see Nowell, 2021). As Flynn et al. (2013: 303) argue ‘two important points that are particularly evident in human populations are that children are not passive recipients of an adult's instruction and that instructors are not always adults … Thus, to a degree, and consistent with NCT, children direct their own learning by shaping their own learning environment’.

This means that, while cumulative human culture is often addressed as a species-wide phenomenon, there is significant lineage-specific variation because different human populations furnish their ontogenetic niches differently. The agency of children as they move through these niches will result in cultural evolutionary trajectories of technological, social and epistemic change that differ in space and time.

## Life-history, cognitive plasticity and object play

The unusual life-history of humans has long been acknowledged (e.g. Key, 2000; Mace, 2000; Thompson & Nelson, 2011). In this context, the significant protraction of the pre-reproductive life stages of infancy, childhood, juvenility and adolescence is particularly noteworthy as they are argued to facilitate the extensive and flexible learning strategies that underwrite human culture (Bogin, 1997, 2006; Högberg & Gärdenfors, 2015; Nowell, 2016). Articulated with this period of extended childhood/adolescence is the notion of ‘extended parenting’ that provides the appropriate niche environment for youngsters to develop cognitively (Uomini et al., 2020) and to be exploratory prior to the onset of reproductive demands (Gopnik, 2020), although these studies do not address the specific role of objects in these ontogenetic niche spaces. Studies of non-human primates have found that social learning is positively correlated with longevity, suggesting that there was selection for increased time for social learning, opportunities to make the most of that learning, and for passing on that knowledge to offspring (Street et al., 2017).

The experimental studies of Iriki and colleagues offer interesting additional insights (Iriki & Sakura, 2008; Iriki & Taoka, 2012). By provisioning captive Japanese macaques – who also use a range of tools in the wild (Leca et al., 2008) – with rakes with which to obtain food rewards, they were able to demonstrate how using such tools actually modifies neural connections within the lifetime of a single individual. Similar neural plasticity has been demonstrated for humans by Mithen and Parsons (2008), whose experiment focused on musical ability, and by Stout, Hecht, and colleagues, focusing on flint tool production (Hecht et al., 2015; Stout & Chaminade, 2007). These studies, working from evolutionary questions, align with numerous advances in the cognitive and medical sciences that have demonstrated how different forms of neural plasticity are fundamental to acquisition, mastery and specialisation in complex motorial, cognitive and social activities (e.g. Blakemore & Frith, 2005; Kolb & Whishaw, 1998; Magee & Grienberger, 2020).

In humans, such activities commonly involve material culture. Malafouris encapsulates the feedback relationship between material culture and cognition in his example of the ‘Blind Man's Stick’, in a striking parallel between the Japanese macaques’ rakes and the most ubiquitous and probably most ancient of human tools, the stick (cf. Oswalt, 1976; Rios-Garaizar et al., 2018). Specifically, Malafouris (2013) asks where a blind man's mind ends and his world begins, arguing that it is at the tip of his cane where the tactile is transformed into the visual. Through this example, he advocates for an extended cognition wherein the mind is not relegated to the skull but instead stretches into the material world.

**Figure 2.**
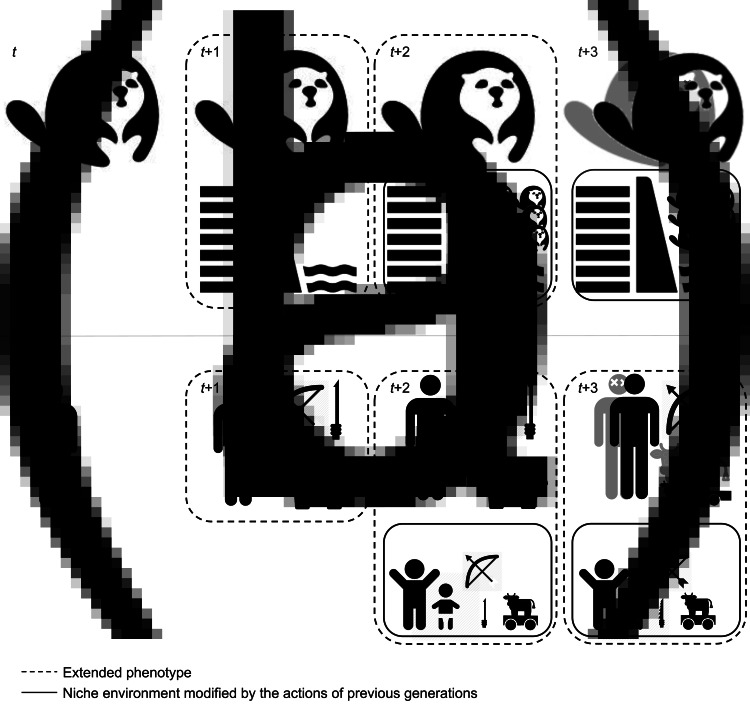
A conceptual model of how niche furnishings change over time within (a) beavers and (b) humans. Prior to any niche construction, the organism interacts with an unmodified environment at *t*, for instance when moving into a new territory. Incipient niche construction begins at *t* + 1, where many of the niche furnishings can also be seen as the extended phenotype of the organism in questions. At *t* + 2, the original organism has offspring that are born into a niche that already is modified, including the ontogenetic environment. These furnishings are no longer extensions of the new generations phenotype but rather part of their modified environment. This feedback-rich relationship continues into *t* + 3 (and *t* + *n*), where the original organism is dead but the niche provisioning continuous, now along specific historical trajectories.

While experimental studies offer valuable insights into the mechanisms of neural plasticity and pruning, they rarely if ever capture such dynamics across multiple generations and in ecologically realistic settings. Processes of neural adaptation respond to social and material cues that channel neural connections into particular, historically and culturally specific forms, which over time may form lineages. The ecologically inherited, constructed niche furnishings serve as combined physical *and* informational resources that condition the formation of these lineages, where the baseline of what constitutes the inherited ecology shifts in each generation (Figure 2).

## Domain-specific innovation through play object priming in Arctic prehistory

Greenlandic prehistory is characterised by a succession of colonisation episodes – the Paleoeskimo (Saqqaq, Independence I/II, Dorset) and subsequent Thule cultures – beginning around 2500 BCE. Although long-lived, the various Paleoeskimo occupations eventually ended around c. 0 CE, albeit with some regional holdouts such as the Late Dorset persisting in some areas until c. 700 CE (Appelt et al., 2016). After a lengthy hiatus from about 1200 CE a new cultural complex appeared in Greenland: the Thule. These later peoples are the direct cultural and biological ancestors of living Inuit peoples today (Raghavan et al., 2014). Over time, Arctic technologies were refined to include sophisticated weaponry (Grønnow, 1994, 2012), instruments, facilities, sledges and different kinds of watercraft. The two cultures practised broadly similar subsistence economies and experienced similar environmental conditions. Yet Paleoeskimo (especially Saqqaq) material culture is ‘remarkably uniform’ throughout their tenure in Greenland (Gulløv et al., 2004: 105) and may even have included the loss of, for instance, bow technology in certain regions and periods. This contrasts with Thule material culture, which included much larger, seaworthy *umiak* boats, but which was also highly dynamic in the development of many diverse harpoon forms, kayak designs and clothing styles – all of which are also pervasively present as play objects (see also Figure 5a–d).

In comparison with the Thule, play objects are scarce in Paleoeskimo contexts. Knuth (1968), for instance, reports the presence of a ‘toy’ ivory harpoon fore-shaft from Independence II contexts, but this identification could be a misinterpretation of the artefact function based singularly on its small size. Appelt and colleagues (2016: 787 and 792) do note the possibility of some miniature tools from Late Dorset contexts, such as unusably tiny soapstone lamps and harpoon heads, as being toys, but they also acknowledge that their exceptional craftsmanship may preclude a purely play-oriented function (but see Langley, 2018). Objects such as miniature weapons and tools, boats and human and animal dolls could have played an important part in the establishment of gender roles and identities (e.g. Fienrup-Riordan, 1983), as well as in the transmission of specific cosmological (i.e. animistic) notions (Fienrup-Riordan, 1994). By the same token, these objects could also have aided in active ecological (e.g. Mithen, 1991; Sugiyama & Sugiyama, 2009) and technological learning and hence guided variation through their inherent mnemonic qualities and as examples of the specific affordances of these complex materials and tools.

**Figure 3.**
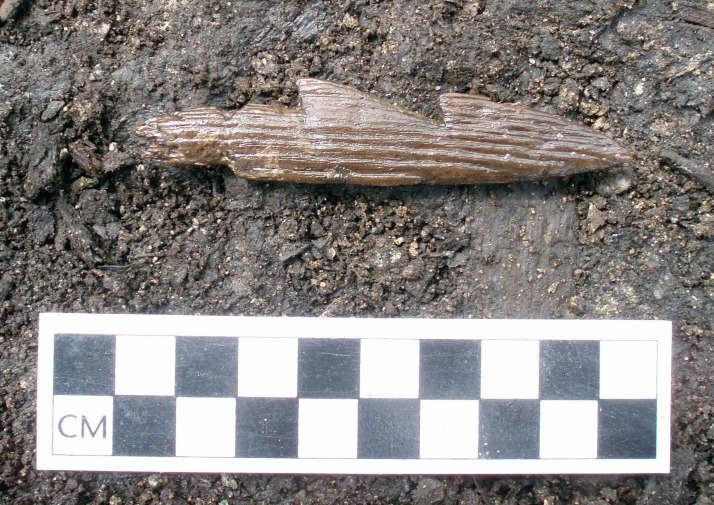
A diminutive harpoon fashioned from wood, from Ainu Creek site, Urup Island, Kuril Islands, Russian Far East. Photo: Matthew J. Walsh.

This point is all the more evident in the astoundingly rich record of children's material culture from Thule sites (Park, 1998, 2005; Park & Mousseau, 2003) and is further reflected in ethnographic reports of children's behaviour and equipment in Arctic societies (e.g. Hawkes, 1916). Thule children can quite readily be identified in the archaeological record through, for instance, their spatial signature (off-site miniature tent rings with pebble meat and fat pieces – see Hardenberg, 2010; and Cory, 2021; Langley, 2020), as well as small but fully functional tools and weapons as well as human/non-human dolls (Figure 3; see also Park, 1998). However, throughout the North American Arctic, it should be noted that ‘dolls’ and figurines – while they certainly served as objects of amusement and learning – also represent other potential functions beyond ‘play’ (see, for instance, Boas, 1964: 157–166), having served in numerous ritual contexts as well (Boas, 1964: 152).

Ethnographically, the presence of such diminutive implements and objects is ubiquitous across the North American Arctic, from Alaska (Birket-Smith, 1953; Gubser, 1965; Nelsen, 1900) across the Canadian Arctic (Balikci, 1970; Boas, 1964; Mathiassen, 1927) and throughout Greenland (Kroeber, 1899; Mathiassen, 1933). Langley and Litster (2018) and Kamp and Whittaker (2020) provide extensive reviews of this literature. As Park (2005) notes, this difference between the miniature record across Paleoeskimo and Thule cultures cannot be readily reduced to differential preservation. These aspects of material culture are usually discussed in terms of socialisation, gender roles and the sexual division of labour. No doubt, play objects are important in this regard. Given the evident correlation between what kinds of miniatures can be identified archaeologically and the technological domains in which Thule cultures are particularly innovative, their role as salient niche furnishings also stands out. For example, miniature hunting tools such as harpoons and bows-and-arrows mirror their adult prototypes and their use by children is ethnographically directly linked to the learning of specific skills (e.g. Laughlin et al., 1991; Losey & Hull, 2019); miniature bows and tiny ‘toy’ harpoon heads are frequently recovered as part of archaeological assemblages (e.g. Larsen & Rainey, 1948). Park and Mousseau (2003) show that miniatures smoothly scale up into adult sizes, reminding us that childhood and adolescence are themselves graded life-stages. The abundant presence of differently sized, adult-manufactured and fully functional miniatures of these complex technologies attests to the consistent niche provisioning in these cultural contexts.

Growing up in these two cultures would have afforded different degrees of innovation potential owing to a greater degree of familiarity with the affordances of specific technologies amongst children and adolescents. Ethnographically, youngsters’ learning in Arctic societies is highly experimental and not subject to significant pedagogic interventions by adults. This absence of formal modes of teaching underlines, we suggest, the critical importance of material culture as pedagogical scaffolds, an observation further underlined by the loss of certain specialised technologies among some Paleoeskimo groups (see Maxwell, 1997; McGhee, 1996; and discussions of implication in Prentiss et al., 2015). From the ethnographic and archaeological evidence, there can be little doubt that object play was vital in the maintenance and refinement of technologies within Thule societies.

## Wheels, vehicles and journeys of life and death in the Eurasian Neolithic

**Figure 4.**
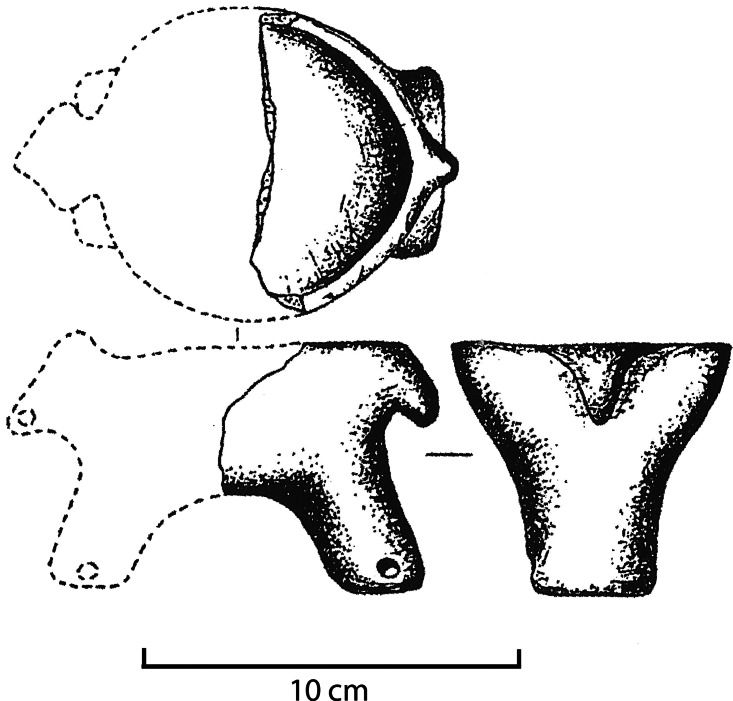
Clay figurine from Late Tripolye context at Karolina, Ukraine. The holes in the legs suggest that this figurine was once wheeled. After Gusev (1998).

Although enshrined in popular culture as perhaps the most iconic invention, the wheel and its application in wheeled vehicles in reality only emerged late in prehistory. The earliest data points for fully fledged wheeled vehicles (vehicle parts, wheel tracks, iconography) are scattered across western Eurasia and all date to the middle of the fourth millennium BCE or slightly later (Burmeister, 2011; Fansa & Burmeister, 2004; Mischka, 2011). Potentially reflecting a rapid spread of this technology, this pattern has left ample room for debate between mono- and polycentric models of its origin (e.g. Sherratt, 1996; contra Vosteen, 1996a, b). Pertinent here is the observation that a miniature application of the wheel and axle combination predates the appearance of cattle-drawn carts and wagons by at least a century: in the fourth-millennium BCE Tripolye contexts of the north-western Pontic region (mainly present-day Ukraine), a range of small, zoomorphic ceramic vessels with holes for two axles have been found (Figure 4). While these presumed axle holes may in principle have served other purposes, their character and location on the figurines are most likely explained by the former presence of axles and wheels made from more perishable materials. These objects are generally regarded as precursors of wheeled vehicles (Maran, 2004; Matuschik, 2006). Conflicting or perhaps complementary interpretations of these as ritual paraphernalia, quotidian objects or playthings abound (cf. Langley & Litster, 2018). Yet, regardless of whether they were designed specifically as play objects or with some other intent, they are likely to have been handled and played with by children.

Although not very precisely dated, it is clear that the apparently wheeled figurines from Tripolye contexts predate the first full-scale wooden wheels and date to a period when these societies saw significant cultural and socioeconomic changes and innovations. During the centuries 4100–3600 BCE, Tripolye settlements developed into proto-urban communities (Menotti & Korvin-Piotrovskiy, 2012; Müller et al., 2016) and a number of significant technological changes occurred: new forms of in-house weaving production, novel techniques of large-scale pottery production employing three-channelled pottery kilns and cattle-drawn sledges for the transportation of materials and goods within, around and to the urban area appear (Kirleis & Dal Corso, 2016; Korvin-Piotrovskiy et al., 2016; Müller & Rassmann, 2016).

The latter technology is particularly significant because the co-existence of an animal-drawn (non-wheeled) form of vehicle and wheeled miniature items presented preconditions for developing full-scale wheeled vehicles. However, the two needed to be combined creatively, and in this process inquisitive and entrepreneurial children and adolescents could plausibly have played a role. Youngsters in this cultural niche had probably observed, handled and played with wheeled objects and thus acquired some familiarity with the mechanical affordances of wheel-and-axle technology, and they would have been equally familiar with the affordances of an animal-drawn transport. Fusing these two sets of experience and translating the combination into a new, operational technology would have required both the ability and openness to associate the two cognitively, and the time, freedom and curiosity to follow up with trial-and-error exploration at full scale. In contrast to Tripolye adults, some youngsters in this context are likely to have simultaneously fulfilled all of these requirements. At the same time, however, youngsters’ interaction with adults was probably key in allowing implementation of innovations in society, requiring both resources and authority held by adults mainly – their tinkering may have laid the foundations for innovations and improvements implemented by adults.

While the origin of full-scale wheeled vehicles in all likelihood lies in the period immediately preceding the middle of the fourth millennium BCE, and while Tripolye is the strongest candidate for the cultural niche in which the development took place, the downstream consequences of this innovation unfolded gradually. It is not until the very end of the fourth millennium that some cultural niches across this vast region are significantly impacted by this technology. From around 3100 BCE, the archaeological finds of wheels and vehicle parts increase dramatically in the Pontic Steppe and in Central and Northern Europe (Burmeister, 2011; Piggott, 1983) and their importance grows significantly, not least in groups that adopt mobile pastoralism around this time (Anthony, 2007; Johannsen et al., 2016; Schroeder et al., 2019).

Interestingly, there follows a secondary process of innovation relating to the wheel, resulting directly from the presence of wheeled transport in the cultural niche. In some groups, the dead are now buried in or with wheeled vehicles, sometimes complete with a team of draught oxen – ‘animal machines’ that were themselves under direct niche-constructing pressures that are observable in their skeletons (Johannsen, 2006, 2007) – indicating that the transition from the world of the living to that of the dead is undertaken by wheeled vehicle. A particularly clear manifestation of this belief is found on the Jutland Peninsula (Denmark), where individual cart burials accumulate into linear cemeteries alongside the roads used by the living (Johannsen et al., 2016; Johannsen & Laursen, 2010). This conceptual innovation relates directly to the affordances of wheeled transport and to the experience of such transport in life, i.e. to the niche effects of this technology. Yet the new journey of death represented in the funerary rituals and structures of these communities is an entirely unintended consequence of the development of wheeled vehicles, underscoring the complexity of downstream (material and cognitive) niche effects of technological innovation on subsequent behaviour and innovation.

## Discussion

The importance of cognitive niche construction for human cultural evolution has long been recognised (Jessen, 2012; Kerr, 2007; Kerr & Feldman, 2003; Pinker, 2010; Sterelny, 2003), including aspects of embodiment and cognition extended through material culture (Malafouris, 2010; Wheeler & Clark, 2008). These discussions tend to address general, species-wide cognitive changes, however, and side-line the specific and historically contingent effects and feedbacks initiated by ecologically inherited material and semantic resources. Recent modelling that includes agent life-history changes strongly supports that age-specific strategic variation across a given organism's life-course can have strong impacts on learning trajectories at the level of the individual and culture change at the population level (Deffner & McElreath, 2020; Fogarty et al., 2019; Miu et al., 2020).

The works presented in the ‘Children & Innovation’ collection provide insightful case studies reflecting heightened attention to both the role of youngsters and the role of material culture in cultural evolution across different disciplines. Lew-Levy et al.'s (2020) review of psychological and ethnographic cases highlights how the contexts of social learning are central to the potential for innovating behaviours among tool-makers and -users. Of equivalent importance is the freedom to play and tinker with little or no interference, to autonomously explore. It seems, somewhat paradoxically, that pedagogy, privacy and play are each crucial drivers of innovative behaviours, especially among younger age groups.

Langley (2020) and Cory (2021) focus on identifying the spaces in which potentially innovative behaviours, specifically amongst children, play out. In recognising children's spaces, those places in which they feel free to explore, imagine and manipulate – not only tools but thoughts and ideas, away from judgement or outside influence – archaeologists may gain novel insights into the evolution of innovative behaviour as not only social and cognitive processes, but spatial processes as well.

Wilkins (2020) highlights social learning contexts in the development of Middle Pleistocene lithics manufacture in Africa. She explores bottom-up processes of learning wherein play and experimentation – learner-driven modes of development – were probably primary drivers of technical skills and subsequent innovation. Rather than knowledge transfer through teaching, early human tool-makers developed skills through emulation and copying (even overimitating) of observed knappers, and reverse-engineering existing technologies. Evidence for diverse core reduction and knapping strategies (i.e. *chaîne opératoire*) and techniques that were used to meet the same final tool type, evince learner-driven technological prowess among tool-makers early on in the development of their knapping skills.

The physical dimensions of growing up are not to be ignored when addressing the role of youngsters in the societies of the deep past. Halcrow et al. (2020) demonstrate the vital role of infant care in survival and nurture, while Nowell and French (2020) explore the concept of adolescence – itself a somewhat ambiguous age category, especially in the archaeological record – and how both the biological and social changes experienced by adolescent individuals may make them more disposed to innovative behaviours or to behaviours prone to lead to innovation (e.g. recklessness and exploration of existing boundaries). Suddendorf et al. (2020) review the human capacity for using containers of various sorts, and specifically the logic of using such meta-tools as perceived and practised by children. Significantly, for this issue's focus on innovation, their observation that ‘recognition of future utility is essentially what turns a problem solution into an innovation’ is astute, as it accounts for the individual temporality that any single innovation must overcome to be recognised as such.

Experimental studies investigating youngsters’ innovation behaviour demonstrate their general capacity for it (Neldner et al., 2020), but only account poorly for the affordance of familiarity amongst the subjects and the actual risks and costs of innovating. The experimental study of Lister et al. (2020) shows how, in laboratory settings, children navigate innovation and enculturation in spontaneous sign innovation. It is here, too, that the works presented in this collection and this paper provide salient insights. Our two cases derive from very different ecological (Arctic, temperate/arid) and societal settings (hunter–gatherers, agriculturalists). Both address domain-specific technological changes that can be plausibly linked to the presence of functional miniatures, and both also demonstrate the rich connections between material culture change and ontologies addressing larger causal relations.

In the later Thule societies in the Arctic, for instance, a niche-constructed learning landscape of guided variation and practice probably facilitated adaptive behaviours such as tinkering, effectively encouraging innovation from an early stage in childhood. The North American Arctic is thus a good example in which a constructed environment populated with toys and spaces to experiment with their use, manufacture and function appears to have provided the opportunity for innovation at small scales. Guided variation seems to have been the principal form of pedagogy throughout the region, exemplified in Balikci's (1970: 105) observation among the Netsilik that ‘[l]earning proceeded exclusively through observation and imitation; no formal teaching whatsoever took place’. This kind of active learning environment probably represents a long-lived social tradition across much of the circumpolar region (Jordan, 2015). In many cases, the flexible expression of active learning has evolved dynamically with socioecological and cosmological understandings, such as notions of rebirth and name-soul traditions which blur the distinction between children, adults and ancestors and the knowledge base that one might have at any given stage of the life cycle (see, for instance, Walsh et al., 2018; Willerslev, 2011). In this sense, not only was the material environment a constructed niche, so too was the social environment and with it the specific learning environment. Under traditional Arctic rebirth belief systems, miniatures were small tools and weapons for small people, who merely needed to remember their use through practice. The implications of such ontological reckonings on play and pedagogy remain unexplored, but could provide valuable insights into the dynamics between uninhibited innovative behaviours and cultural transmission processes. Albeit situated further back in time, and firmly outside the range of ethnohistorical information, our archaeological case from the fourth millennium BCE also bears witness to the interplay between material affordances, technologies and understandings of cause and effect. Detailed studies on remarkable materials such as the figurines from Mal'ta by Lbova (2021) hint at how far back in time we may be able to bring such perspectives.

**Figure 5.**
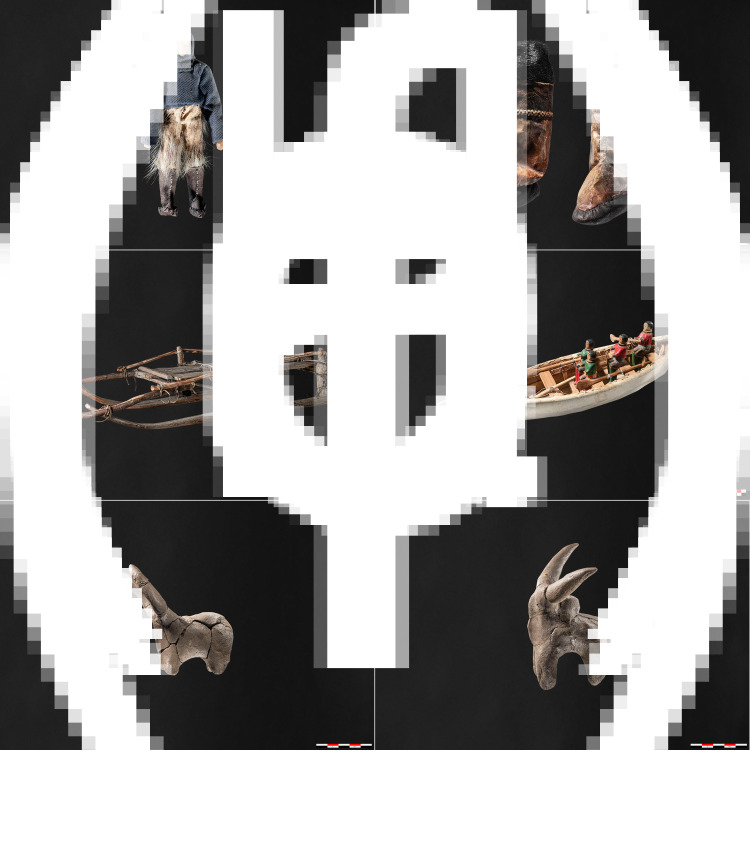
Examples of miniatures from Arctic (Inuit, a–d) and tropical (Wodaabe, e–f) contexts from the collection of Moesgård Museum, Denmark. All of these objects were manufactured by adults for children. The clay figurines have close parallels in archaeological contexts as ancient as ~17,500–15,000 years (cf. Farbstein et al., 2012) as well as in many later prehistoric examples. Examples a–d are closely related to the miniatures discussed in our Paleoeskimo case study. Do note how many of the materials used are highly perishable, making the detection of such objects in archaeological contexts challenging.

Capped by a concluding discussion by Sterelny (2021), the studies presented in this collection strongly support the notion that using and thinking with material culture constitutes an important binding element between social learning strategies, life-histories, external environmental cues and innovation (Johannsen, 2010, 2014). This perspective has important implications for our understanding of human cultural evolution. First, major biological life-differences exist between *Homo sapiens* and many pre-modern hominins. These may have fundamentally constrained learning opportunities and the material culture-mediated affordance familiarity we have demonstrated here (Nowell, 2016; Nowell & White, 2012). In addition, an interesting pattern is also emerging from the archaeological record: there is, at present, suggestive evidence for miniatures early in human evolution (Assaf, 2021; Stapert, 2007). The number and range of such objects expanded dramatically in the Upper Paleolithic of Europe (Farbstein et al., 2012; Langley, 2018; Langley & Litster, 2018; Pfeifer, 2015) and the Americas (Ellis, 2004; Guarino & Sellet, 2019), and further in the Neolithic (e.g. Carter, 2006). Many of these objects find close matches in the ethnographic record (Figure 5). As with virtually all prehistoric miniature artefacts, it will be difficult or impossible to ascertain beyond any doubt their intended principal function (cf. Crawford, 2009); some miniatures surely did belong in ritual contexts, but the evident fluidity between quotidian and sacred contexts in pre-modern societies – and the obvious presence of children at all times in human prehistory – also makes such distinctions somewhat moot (cf. Langley & Litster, 2018). Alongside specific social settings conducive for innovation (Lew-Levy et al., 2020), the furnishing of ontological niches with miniatures offers a potential mechanism for how and why we see an increase in innovation rate in certain periods and technological domains.

## Conclusion

The collection of papers we introduce here powerfully supports the notion that object play was an important element of human cultural evolution and that play objects served as vital cognitive and pedagogical scaffolds for material culture innovation. We do not deny the importance of expertise nor of adults actively seeking innovative solutions. We do argue, however, that experimentation through object play may be an overlooked novelty-generating mechanism in obligate tool-users such as *Homo sapiens*. This dynamic is amplified through provisioning of human ontogenetic niches with miniatures; and these dynamics are predicted to vary in space and time, but also across individual life-history stages, as young children play very differently to almost mature subadults.

In taking such insights further, we urge experimentalists to devise tasks that incorporate affordance familiarity rather than to divorce it from them. In addition, we would welcome tasks that investigate the link between material culture and narrative cognition where causal relations in the latter are inferred from analogues in the former. *In silico* models could be set up in such a way as to track not just cumulative culture per se but the accumulation of multiple local optima (e.g. Caiado et al., 2016) and their contingent specifics. Just as agents can be designed with different properties, perhaps such models could also be designed to let material culture play a more active part in the evolutionary process.

The ethnographic record offers a plethora of resources for further investigating the hypothesis presented here. Drawing on rich cross-cultural resources such as HRAF (see Ember & Cunnar, 2015), it would be possible to systematically and quantitatively interrogate the relationship between who made what play objects, whether their complexity increases with age, or whether it is primarily adults who manufacture functional miniatures for children. There is much potential in approaching play objects using the same analytical protocols that are applied to adult material culture (Haidle, 2014; Perreault et al., 2013). Such basic research would allow us to assess and track if and how changes in youngsters’ objects can be observed on par with their adult counterparts. In turn, classificatory insights gained from the investigation of ethnographic materials as well as the countless miniatures held in museum storerooms (see Figure 5) can then be used to re-visit the archaeological record in search of play objects. These data could also be used directly to test the suggested correlation between play objects and innovation rates. One striking conclusion of the cases discussed here is that most innovation appears to occur in past societies with evidence of *both* highly consistent learning processes *and* a rich array of children's material culture (e.g. the Magdalenian, the Thule, certain parts of the Eurasian Neolithic).

The papers forming the special collection on children and innovation enrich our understanding of the role of youngsters in cultural evolution, and so of cultural evolution at large. They show how experimental, ethnographic and archaeological sources can be marshalled to provide new insights into the embodied mechanics of learning and cultural transmission. Clearly, we need to take the changing abilities and agency of small but growing humans more fully into account when trying to understand the patterns and processes of cultural evolution.

## Research transparency and reproducibility

No data are associated with this article.
